# Elucidating the relationship between dyslipidemia and osteoporosis: A multicenter, prospective cohort study protocol

**DOI:** 10.3389/fcvm.2022.901786

**Published:** 2022-09-14

**Authors:** Xu Wei, Yili Zhang, Chuanrui Sun, Baoyu Qi, Xinyi Huang, Ming Chen, Ning Liu, Kai Sun, Xin Chen, Xiaokuan Qin, Yanming Xie, Liguo Zhu

**Affiliations:** ^1^Wangjing Hospital, China Academy of Chinese Medical Sciences, Beijing, China; ^2^Institute of Orthopaedics of Beijing Integrative Medicine, Beijing, China; ^3^School of Traditional Chinese Medicine & School of Integrated Chinese and Western Medicine, Nanjing University of Chinese Medicine, Nanjing, China; ^4^School of Traditional Chinese Medicine, Beijing University of Chinese Medicine, Beijing, China; ^5^Institute of Basic Research in Clinical Medicine, China Academy of Chinese Medical Sciences, Beijing, China

**Keywords:** osteoporosis, dyslipidemia, prospective cohort study, follow-up, study protocol

## Abstract

**Background:**

A previous study has shown similar factors in dyslipidemias (DL) and osteoporosis (OP). However, no cohort study has been reported on the association between DL and OP in the postmenopausal population in China. This study aims to provide epidemiological and pathophysiological evidence regarding the association between DL and bone mass and fracture risk.

**Methods:**

This is a multicenter, prospective cohort study that will have approximately 1,100 representative participants enrolled from multiple hospitals or communities in China. They will be divided into two groups according to whether or not they are exposed to dyslipidemia and will be epidemiologically investigated. Each participant will be visited continuously once every year with a minimum follow-up of 3 years to track incidences of OP. Meanwhile, free bone density screening, questionnaires, and blood sample collection will also be completed during this period.

**Conclusion:**

The current study is likely to provide greater insight into the relationship between lipid metabolism and bone metabolism in postmenopausal women. Furthermore, the research result maybe fed into public health strategies with regard to metabolic disease prevention.

## Introduction

Osteoporosis (OP) is an escalating public health issue that affects more than 200 million people worldwide, with an increasing prevalence in elderly people (1). According to Chinese authoritative data, OP has become an important health problem for people over 50 years old in China, and the problem of OP is particularly serious in middle-aged and elderly women. About 32.0% of people over 65 years old have OP prevalence, of which 10.7% are men and 51.6% are women (2). Dyslipidemias, including a wide range of disorders in lipoprotein metabolism, constitute one of the leading causes of atherosclerotic cardiovascular disease worldwide (3). In addition, patients suffering from DL have an increased risk of metabolic syndrome and obesity (4), which are important causes of premature death and decreased disability-adjusted life-years (DALYs) worldwide (5).

From 2017 to 2020, we conducted the community-based OP and associated fractures screening in the Beijing area (BEYOND study) in order to explore the prevalence of disease, risk factors, and the characteristics of related biomarkers (6, 7). In this study, we found that leptin appears to be a key biomarker associated with PMOP and in combination with other indicators can predict the occurrence of PMOP, suggesting a potential association between lipid metabolism-related indicators and OP (8). Given the worldwide increased body mass index (BMI) and exponentially growing obesity rate, understanding the risk of osteoporosis in the DL population has never been more important (9). Furthermore, recent studies have also increasingly provided evidence of a strong relationship between dyslipidemia and osteoporosis (10, 11). It is suggested that every osteoporotic patient should be screened for dyslipidemia (12).

A previous study has shown similar factors in DL and OP such as lifestyle, inheritance, metabolism, nutrition, and hormone (13). To the best of our knowledge, no cohort study has been reported on the association between DL and OP in the postmenopausal population in China. Hence, a sequential exploration of the deeper causal relationship between DL and OP can provide new evidence for the co-management of these two diseases. We further designed the multicenter prospective study called “DyslipidemIA and risk of osteoporosis in postMenopausal population: a multicenter, prospective cohort stuDy (DIAMOND study),” to provide epidemiological and pathophysiological evidence regarding the association between DL and bone mass and fracture risk. On the contrary, we still keep the expectation that this study will provide the latest evidence for the research of different metabolic diseases.

## Methods

### Study design and settings

The multicenter, prospective cohort study aims to explore the clinical associations between the DL and OP in Chinese postmenopausal women. Data will be collected from a representative sample and the participants will be recruited from multiple hospitals and communities in China. Then, the subjects will be divided into two groups according to whether there is dyslipidemia or not, and an epidemiological investigation will be conducted.

**Figure 1:**
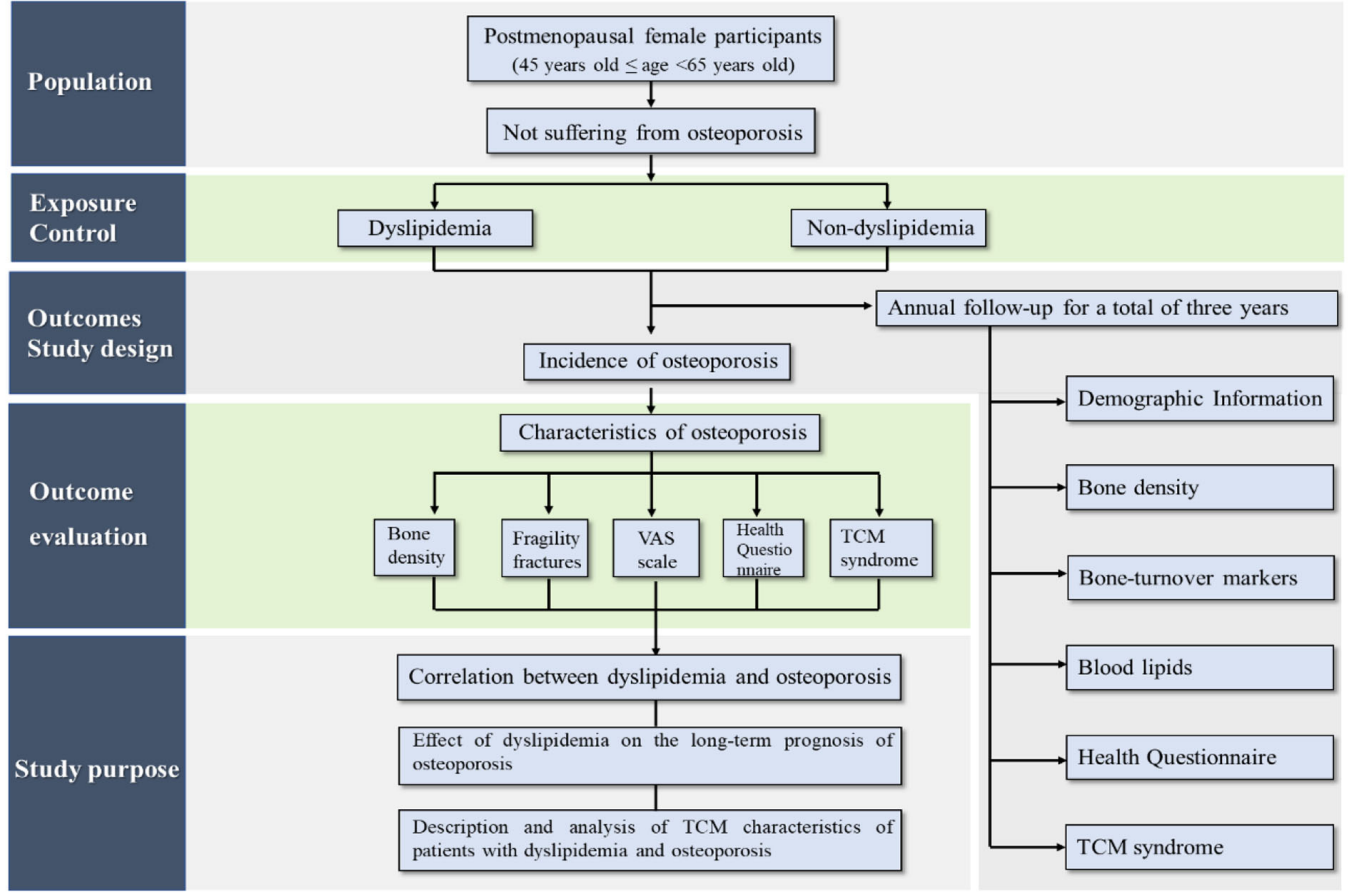
Flow diagram of the study.

Each participant will be visited continuously once every year with a minimum follow-up of 3 years to track incidences of OP. Furthermore, BMD screening and biological samples will also be gathered. The questionnaire will be administered to collect information about personal characteristics such as health status and potential risks. Important information including the incidence of OP and/or fracture and medication use will be confirmed annually during the follow-up period. The required sample size will be at least 1,100 patients based on a power analysis. A flow chart of the research process is shown in Figure 1.

### Ethics and registration

This study was approved by the medical ethics committee of the Wangjing Hospital, China Academy of Chinese Medical Sciences (approval number: WJEC-KT-2021-040-P002) on 31 August 2021 and registered in the Chinese Clinical Trial Registry (approval number: ChiCTR2100051370) on 21 October 2021. The participants will be told the purposes and trends of the research, and their rights will be emphasized; then they will be asked to sign an “informed consent form” in accordance with the Helsinki Declaration.

### Participants

Due to the potential impact of different socioeconomic conditions on population bone mass, participants in this study will be recruited in different regions of mainland China (namely, northern China, southern China, and central China) from October 2022 to May 2023. The aim is to avoid the potential bias caused by economic circumstances, education, work, and other factors.

The subjects will be enrolled according to the established eligibility criteria: (1) Post-menopausal women; (2) aged from 45 to 64 years old; (3) *T-*value of bone density measurement is >-2.5; and (4) the participants are willing to participate in the investigation study.

Restrictive exclusion criteria are defined as follows: (1) A fragility fracture is found in the enrolled female; (2) the population who suffers from mental illness or are unable to complete the study.

### Exposure and control

We will divide the participants into two groups according to whether they have dyslipidemia, which means that dyslipidemia is an exposure factor in this study.

### Outcomes of interest

The pre-specified outcomes will be observed in detail and the outcomes reflecting bone transition are described. The primary outcome is the incidence of OP. During the study period, the condition of normal bone mass, osteopenia, and fragility fracture for each female will be also identified every year. For the event of OP-related fracture, different sites, occurrence times, and the number of fractures are completely recorded.

The secondary outcomes are radiographic BMD assessment and serum marker test. The blood indexes aim to reveal the dynamic changes in calcium and phosphorus metabolism, osteogenesis, and osteoclast function. Also, the recognized scales such as the health question scale, and specific relevant TCM syndrome differentiation scale will be investigated.

### Questionnaire information

Face-to-face structured interviews will be done with a well-designed questionnaire by trained interviewers. The questionnaire will be used to collect information on the five collected domains. The aspects include: (1) general demographic information; (2) lifestyle and dietary behavior; (3) health status, comorbidities, and medication-taking condition; (4) recognized scales measuring osteoporosis risk, physical activity or quality of life; and (5) characteristics of TCM syndromes.

All the subjects with the researchers will complete the questionnaires at baseline and follow-up phase. Then, a complete post-enumeration check of the questionnaires will be performed.

### BMD measurement

The BMD values can be measured at multiple skeletal sites using the accepted dual-energy x-ray absorptiometry (DXA) scanner manufactured by Hologic Inc. In the absolute BMD measurement of the human body, it is necessary to record the information of the lumbar spine, both hips, and the distal one-third of the radius site. In addition, the total *T-*value and the *T-*value of each site will be reported separately. Definitions of osteopenia and OP are in accordance with WHO standards on BMD measurement.

### Laboratory test

Each participant will be required to complete blood specimen collection in the fasting state. In total 10 ml of blood sampling will be collected through intravenous vacuum blood method after a nurse checks the personal information. Then the samples will be centrifuged and cryopreserved according to the standard operating procedures.

The indicators will include serum alkaline phosphatase, osteocalcin, bone-specific alkaline phosphatase, serum type I procollagen C-terminal peptide, serum type I procollagen N-terminal peptide, and 25OHD. Biochemical indicators such as liver and kidney function, plasma glucose, total cholesterol, triglycerides, high-density lipoprotein, low-density lipoprotein, and uric acid will be also tested. Meanwhile, the BMD measurement and laboratory tests annually will be cost-free in the process of study.

The Guangzhou KingMed Diagnostics Limited Liability Company will be responsible for collecting, transporting, and testing blood samples. Samples that cannot be sent in time for testing will always be stored in a 4°C refrigerator (within 6 h); if the sample cannot be sent on the day of collection, the serum will be centrifuged (centrifugation conditions: 2,500 ~ 3,000 rpm, 4°C, 5 min) and retained and frozen in a −20°C refrigerator until sent for testing. Blood samples will be kept at −20°C during all transport processes.

### Follow-up program

All of the participants will be followed up by face-to-face interviews as far as possible. In the follow-up survey, we will conduct in the same way as the baseline investigation. For mobility-impaired fracture patients in some cases, a survey with options for telephone interview may be considered.

First, the detailed information on the telephone number of the subject or their relatives and clear home address will be noted down. Second, the researcher in-charge will contact the participants ahead of time on both workdays and weekends to ensure they can participate in the follow-up every year. Third, the self-reported disease status and medication use will be collected through telephone interviews or household surveys when the on-site follow-up is not available. For the subjects who could not be investigated face-to-face or contacted by telephone surveys, medical records in designated hospitals or community healthcare centers will be acquired possibly to confirm their health status. The expected proportion of participants lost to follow-up is below 10%.

### Sample size calculation

We calculated that a minimum sample size of 1,100 (550 DL and 550 non-DL) women are required to demonstrate an 8% difference in the prevalence of OP between DL and non-DL participants (14). The assumes are as follows: best-estimate OP prevalence of 12% among non-DL women and 20% among DL women; 5% level of significance; 90% power for a two-sided test. Furthermore, we considered a 20% dropout rate and finally calculated the sample size.

### Data management

The stepwise data management process is designed for implementation in this study. The electronic data will be input by the two independent assistants through the Epidata database. All the information will be confirmed eventually after the consistency check of the data are completed. During the follow-up period, clinical data management will be developed based on the needs of predetermined outcome research, including the main outcome measure and updated examination results.

### Statistical analysis

Statistical analyses were conducted by using SAS 9.4 (SAS Institute, Cary, NC) and R software 4.1.1 (R Foundation for Statistical Computing, Vienna, Austria). Figures were created in GraphPad Prism 8.0 (GraphPad Software, CA, USA). The continuous variables which are in accord with normal distribution will be expressed as the mean and standard deviation, while the measurement data that do not coincide with normal distribution will be expressed as the median and interquartile range. In the description analysis, the *t-*test or the Mann–Whitney *U*-test will be used for comparing quantitative data between the two groups. On the contrary, the count data will be presented as frequency and percentage, and the χ^2^-test will be applied for the comparison between the two groups.

The incidence of OP in two groups will be counted. Generalized estimation equations will also be designed to confirm the effect of potential risk factors on OP. A non-adjusted model and a multivariate-adjusted model will be utilized, and the results will be reported using unadjusted, minimally adjusted, and fully adjusted analysis, according to the specifications of the STROBE statement. A two-sided *alpha* of 0.05 will be considered to be statistically significant.

## Discussion

Osteoporosis is a metabolic bone disease which may coexist with other metabolic diseases such as dyslipidemia, obesity, diabetes, non-alcoholic fatty liver disease, and cardiovascular disease. An intriguing link between lipid abnormalities and bone metabolism was demonstrated in patients with cardiovascular disease (15). Even more importantly, some studies revealed that DL was negatively correlated with OP, and the effect of cholesterol on bone metabolism depended on its different types (16). At present, the shared pathogenic mechanisms or even cross-talk effect between bone metabolism and lipometabolism may also exist (17). An important role in the regulation of lipid metabolism is attributed to the estrogen secretion in postmenopausal women (18, 19). Menopausal women are more likely to form fat accumulation, with an increase of total cholesterol, triglyceride, LDL-C, and decrease in HDL-C (20). High cholesterol is involved in the cell functions of bone tissue, and it has recently been shown to increase osteoclast activity and decrease osteoblast function (21). There are some molecules involved in the interaction between bone metabolism and lipid metabolism, namely, osteoprotegerin (OPG) (22), apolipoprotein E (APOE) (23), peroxisome proliferators-activated receptor γ (PPARγ) (24), and vitamin D receptor (VDR) (25).

Overall, the correlation between DL and OP was confirmed preliminarily on the basis of the clinical and fundamental researches. However, whether there is a causal relationship between DL and OP or not is not fully revealed. Furthermore, the potential or deeper relationship between DL and OP in postmenopausal women is of great clinical significance in the co-morbidity study, guiding drug selection, and developing new treatment schemes.

Our study has highlighted the scope for inclusion of multicenter, which enables these results, can be extrapolated effectively to other areas. To our knowledge, it is a large population-based longitudinal cohort study for this purpose in China. Besides, the current cohort will not only focus on traditional lifestyle-related and sociodemographic factors, but also conduct comprehensively research on important comorbidities, medication management, risk of fracture, etc. Another feature is the integration of clinical information, BMD assessment, and serum marker test. Through the observation of long-term follow-up, the dynamic change of bone and bone turnover markers are recorded completely. Most important of all, the potential causal relationship and the effect of DL on bone metabolism may be objectively evaluated.

However, there are several limitations in our cohort study. First, the survey sites and the participants will not be randomly chosen. This is mainly because the units with various regions and the basis for cooperation will be considered in this study to better elucidate the pathogenic mechanism of DL and OP. Moreover, this cohort study is more concerned with the proportion of participants who could be followed up. Thus, we will choose stable communities, hospitals, and participants to ensure a greater follow-up rate. Second, the combined disease surveys in the questionnaire may be biased as a result of the self-reported data, especially not inaccessible through electronic medical records.

## Conclusion

Based on the prospective cohort study and long-term follow-up, the DIAMOND study is likely to provide greater insight into the relationship between lipid metabolism and bone metabolism in the postmenopausal women. Furthermore, the research result maybe fed into public health strategies with regard to metabolic disease prevention.

## Author contributions

LZ, YX, XW, and YZ are in charge of the conception and study design. CS, BQ, XH, MC, NL, KS, XC, and XQ participate in the execution, acquisition of data, quality control, analysis, and clinical interpretation. XW and YZ take part in drafting, revising or critically reviewing the article. All authors made a significant contribution to the manuscript, has agreed on the journal to which the article has been submitted, and agree to be accountable for all aspects of the work. All authors contributed to the article and approved the submitted version.

## Funding

This study was funded by the Innovation Team and Talents Cultivation Program of National Administration of Traditional Chinese Medicine (Grant no. ZYYCXTD-C-202003), the Fundamental Research Funds for the Central Public Welfare Research Institutes (Grant nos. ZZ13-YQ-039, 2020YJSZX-4, and CI2021A02013). In addition, this work was also financially supported in part by the grants from a project funded by the Priority Academic Program Development of Jiangsu Higher Education Institutions (PAPD).

## Conflict of interest

The authors declare that the research was conducted in the absence of any commercial or financial relationships that could be construed as a potential conflict of interest.

## Publisher's note

All claims expressed in this article are solely those of the authors and do not necessarily represent those of their affiliated organizations, or those of the publisher, the editors and the reviewers. Any product that may be evaluated in this article, or claim that may be made by its manufacturer, is not guaranteed or endorsed by the publisher.
